# Usefulness of Early Genetic Diagnosis for Twins With a Family History of Congenital Nephrotic Syndrome

**DOI:** 10.7759/cureus.36667

**Published:** 2023-03-25

**Authors:** Yukiko Toya, Ken Ishikawa, Taro Yoshida, Atsushi Matsumoto, Manami Akasaka, Kandai Nozu

**Affiliations:** 1 Department of Pediatrics, Iwate Medical University, Iwate, JPN; 2 Department of Pediatrics, Kobe University Graduate School of Medicine, Kobe, JPN

**Keywords:** congenital anomaly, early diagnosis and treatment, genetic test, finnish-type congenital nephrotic syndrome, twin pregnancies

## Abstract

We reported a dichorionic diamniotic placental twin (DD twin) with a family history of a congenital nephrotic syndrome of the Finnish type (CNF), of which the parent had heterozygous for the* NPHS1* gene mutation.

The DD twin was born at 36 weeks gestation, and their fused placenta weighed 1,340 g. Although the first-born child had heavy proteinuria and hypoalbuminemia and needed daily albumin replacement to manage severe edema, the second had only mild proteinuria after birth.

Genetic testing performed 28 days after birth detected homozygous for the *NPHS1 *gene mutation in only the first-born child but not in the second, which resulted in performing invasive left nephrectomy and peritoneal dialysis (PD) to manage edema in the first.

For DD twins with a family history of CNF, prenatal diagnosis of CNF may be difficult. Therefore, close postnatal clinical observation and early genetic testing are essential for the diagnosis of CNF.

## Introduction

Nephrotic syndrome within the first year of life is classified into two categories: congenital nephrotic syndrome (CNS), which develops within the first three months of life, and infantile nephrotic syndrome, which develops after that [1]. Although CNF of the Finnish type is a major CNS disease and an autosomal recessive disorder, which occurs in 1 in 8,200 births in Finland [2,3], the incidence of cases in Japan is unknown. More than 200 mutations have been identified in the gene responsible for CNF, *NPHS1* [4].

Nephrin, a gene product of *NPHS1*, exists in the slit membrane between the foot processes of visceral glomerular epithelial cells and plays a role as a signaling molecule, such as in forming filtration barriers [5,6]. In vitro studies have shown that Nephrin is normally expressed in the endoplasmic reticulum. However, in cases of *NPHS1* gene mutations, it is absent on the cell surface, which inhibits its transport to the cell membrane and causes protein leakage from the glomerulus.

A large amount of protein leakage from the glomerulus already occurs during the fetal period, resulting in increased alpha-fetoprotein (AFP) in amniotic fluid [7] and an enlarged placenta [8]. Most patients with CNF develop CNS within the first week of life; without optimal treatment, CNF is fatal. Steroids and immunosuppressants, commonly used to treat ordinary nephrotic syndrome, are ineffective, and the disease is difficult to manage. Reducing massive protein leakage from the glomerulus by removing the kidney requires subsequent blood or peritoneal dialysis (PD); kidney transplantation is the only effective treatment [9,10]. Early diagnosis of CNF and long-term strategies for maintaining patients after kidney transplantation are required to achieve a good prognosis.

We report a case of dichorionic diamniotic placenta twins (DD twins) with a family history of CNF, who required early diagnosis and intervention for rescue and to achieve a good prognosis.

## Case presentation

The patients were twin girls born at 36 weeks and 3 days of gestation. The first-born child's birth weight was 2,179g and the second-born child's birth weight was 2,180g.

Family history

The mother was 32 years old and had a history of two pregnancies with only one birth. The older brother was clinically diagnosed with CNS on the second day of life. After kidney removal and PD, he underwent a living-donor kidney transplant from his father at the age of three. He underwent a genetic test at age 1. CNF was diagnosed by identifying a deleted cytosine at the 2515th base of exon19 on the long arm of chromosome 19, which is the region responsible for the NPHS1 gene. A frameshift mutation (Ex 19 2515delC) was found to stop translation at the 839th amino acid Gln, and a heterozygous mutation was detected in the patient's parents. Currently, the sibling is 10 years old, 140.2 cm tall, weighs 32.2 kg, and shows normal development.

Perinatal period

At 10 weeks of gestation, fetal ultrasonography revealed DD twins. Since the child from the mother's previous pregnancy had CNF, the parents received genetic counseling at our clinical genetics department; however, the parents had a strong desire to continue the pregnancy and did not perform AFP assessment or genetic testing in amniotic fluid to detect the possibility of fetal CNF. An enlarged placenta and polyhydramnios were absent during the fetal period. The twins were born at 36 weeks and three days of gestation by emergency cesarean section due to premature birth.

Physical finding at birth

The twins had fused placentas which weighed 1,340 g. The first-born child was a girl with a birth weight of 2,179 g (-1.0 SD), and Apgar scores were 8 at 1 minute and 9 at 5 minutes. The second-born child was also a girl with a birth weight of 2,180 g (-1.0 SD), and Apgar scores were 8 at 1 minute and 9 at 5 minutes. Neither child had any external malformations, distended anterior fontanelles, or edema, and their respiratory and circulatory functions were stable. Blood and urinalysis at birth revealed hypoalbuminemia and heavy proteinuria (Table 1), in addition to a 2 mm membranous ventricular septal defect in the first-born child. The second-born child had mild proteinuria without hypoalbuminemia (Table 1).

**Table 1:** Blood and urine analysis of both children. The first-born child had marked hypoproteinemia and proteinuria. On the other hand, the second-born child had mild proteinuria but no hypoproteinemia.

Investigation	First-born child	Second-born child	Reference
WBC	11,990	9,500	5,700-18,000 /mm^3^
Hemoglobin	15.8	14.6	13.2-22.0 g/dl
Platelets	28.3*10^4^	37.4*10^4^	8-35.6*10^4 ^/mm^3^
Total protein	2.1	4.6	5.6-8.5 g/dl
Serum albumin	0.9	3.2	3.3-4.5 g/dl
Serum creatinine	0.45	0.9	0.3-0.9 mg/dl
Blood urea nitrogen	14	15	4-20 mg/dl
Lactate dehydrogenase	496	548	364-1,120 U/L
Immunoglobulin G	32	574	1,031±200 mg/dl (mean±SD)
Fibrinogen	437	242	190-420 mg/dl
Antithrombin	13	44	14-62 %
Urinary protein/Urinary creatinine	32	1.1	<0.5

**Figure 1:**
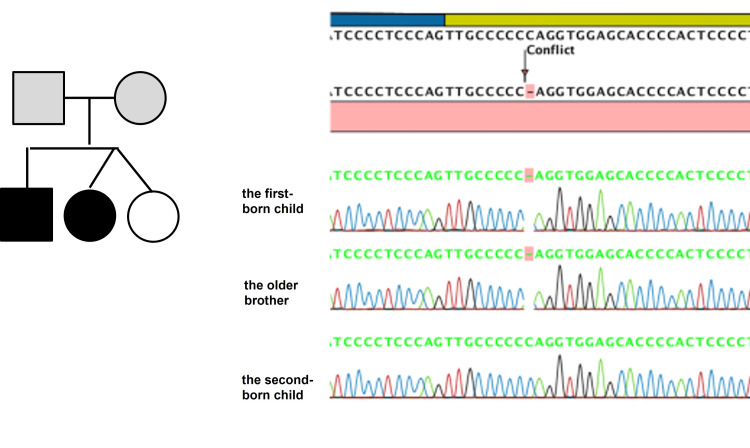
Family tree and genetic test results. Heterozygous mutations were found in both parents and the same homozygote in the brother and the first-born child. The second-born child had none of the mutations.

Since the first-born child was suspected of having CNF and the second-born child also had mild proteinuria, genetic testing of both children was performed on the 28th day after birth. Informed consent was obtained from the parents. At 77 days after birth, the results revealed that the first-born child had the same homozygous gene mutation identified in the previous pregnancy. A definitive diagnosis of CNF was made (Figure 1). There were no gene mutations in the second-born child (Figure 1). 

Postnatal course of the first-born child

**Figure 2:**
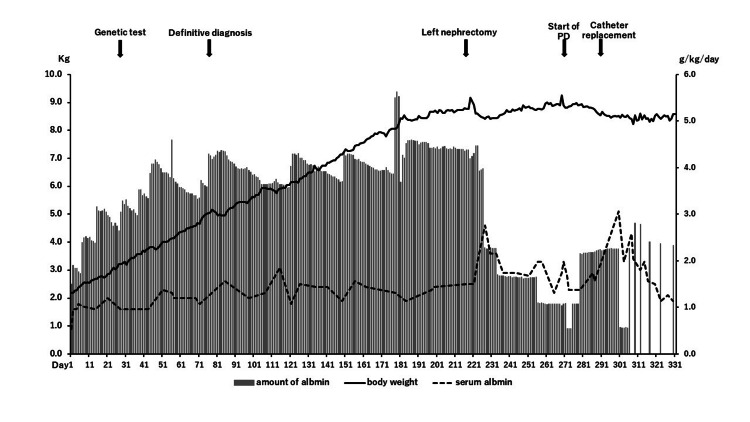
Postnatal course of the first-born child. Daily albumin infusions were performed to maintain serum albumin levels at 2 g/dL or higher. After the left kidney was removed, the amount of albumin infusion was gradually reduced, and the serum albumin levels were maintained by weekly supplementation.

Hypoalbuminemia and severe proteinuria persisted (Table 2). Albumin was administered daily from the first day of life to maintain a serum albumin level of ≥ 2 g/dL and ameliorate edema due to hypoalbuminemia (Figure 2).

**Table 2:** Serum albumin and proteinuria after birth. The first-born child had hypoproteinemia and severe proteinuria. The second-born child had mild hypoproteinemia and proteinuria. UP: Urine protein; UC: Urine creatinine.

Investigation	First-born child	Second-born child	Reference
Serum albumin Day 0	0.9	3.2	3.3-4.5 g/dl
Serum albumin Day 1	1.6	3.4	
Serum albumin Day 2	1.6	3.5	
Serum albumin Day 3	1.8	3.6	
UP/UC Day 0	31.9	1.1	<0.5
UP/UC Day 1	42.6	0.9	
UP/UC Day 2	120.6		
UP/UC Day 3	372.5		

As oral feeding gradually worsened, tube feeding with high-protein low-salt milk and breast milk was administered. Thyroid hormone, immunoglobulin, and vitamins, which leaked into the urine, were replaced, and anti-platelet drugs were administered to avoid thrombotic complications due to CNS-induced hypercoagulation. Although angiotensin-converting enzyme inhibitors and indomethacin were administered to reduce massive proteinuria on the 44th and 124th days after birth, respectively, the effects were insufficient. Therefore, a left nephrectomy was performed to reduce massive proteinuria on the 218th day after birth, when the definitive diagnosis of CNF was made, and PD was started on the 266th day after birth. As a result, the frequency of albumin replacement was gradually reduced, and eventually, edema was managed by once-weekly administration of albumin. The patient was discharged from the neonatal ICU 11 months after birth and continued daily PD. Because of the leakage of immunoglobulins into the urine and subsequent frequent infections, replacement of immunoglobulins more than once a month was needed in addition to albumin replacement at the outpatient pediatric clinic. At three years and three months of age, the right residual kidney was removed to prepare for kidney transplantation, which caused urinary leakage and subsequent frequent replacement of immunoglobulins and inhibited vaccination-instigated antibody acquisition. After removing the right kidney, fluid status was managed by a hybrid of PD and hemodialysis (HD) due to the deterioration of peritoneal function. Living-donor kidney transplantation was performed by her grandmother at four years and four months of age. The patient is 93 cm tall, weighs 12.1 kg, and shows normal development.

Postnatal course of the second-born child

The second-born child showed mild proteinuria and hypoproteinemia after birth (Table 2). Proteinuria gradually decreased without edema, and the patient was discharged from our hospital on the 10th day after birth. After being discharged, she underwent monthly urine tests until proteinuria disappeared and the results of the genetic test were revealed. The second-born child showed normal development at four years and four months of age.

## Discussion

Hypoalbuminemia with heavy proteinuria and mild proteinuria was observed in the first-and second-born DD twins, respectively, with a family history of CNF. A genetic test performed 28 days after birth revealed homozygosity for the *NPHS1* gene mutation in the first-born child but showed no abnormalities in the second-born child. Although daily albumin replacement was needed to ameliorate the edema, left nephrectomy and PD after obtaining a positive genetic diagnosis minimized the need for albumin replacement from daily to weekly. The first-born child was eligible for discharge from the hospital. In the second-born child, proteinuria gradually disappeared, and medical management was not necessary after the genetic testing was performed and a negative diagnosis was made.

CNF is a disease characterized by autosomal latent inheritance. Therefore, in cases where each parent has a heterogeneous abnormality in the *NPHS1* gene, the probability that their child will have CNF is 25%. Most patients with CNF have heavy proteinuria and severe hypoproteinemia within the first few days of life, and in the absence of interventions, it can be fatal. Although an antenatal diagnosis of CNF is available in cases of a family history of CNF [11,12], there is not enough antenatal diagnosis of CNF in Japan. In fetuses, CNF can be predicted by measuring AFP in maternal blood and amniotic fluid at 15-20 weeks of gestation [7] and by the presence of an enlarged placenta [8]. However, the false-positive result that AFP is increased even in children who are heterozygotes of the *NPHS1* gene mutation [13] is not specific to CNS. In this case, each parent had a heterogeneous abnormality in *NPHS1*. Therefore, each twin had the possibility of CFN, but an antenatal diagnosis was difficult. In the case of twin fetuses, it is impossible to distinguish whether each or both fetuses suffered from CNF by measuring maternal blood AFP and placental weight. Further, there are risks associated with collecting amniotic fluid for measuring AFP. The fetal loss rate after amniocentesis in a single fetus is reported to be 0.4-0.5% and 3.0% in twins. Among the positive fetal screenings, there were no differences in the fetal loss rate with or without amniocentesis, which is 8.8% and 6.8%, respectively. On the other hand, the fetal loss rate is higher in the presence of a fetal abnormality [14]. In addition, in the case of twins, there are technical difficulties that could risk puncturing each amniotic cavity.

In children with CNF, kidney function is gradually lost and eventually develops into end-stage kidney failure. Therefore, unilateral or bilateral nephrectomy is performed to ameliorate edema and is accompanied by PD or HD, even when the kidney function is maintained. Since introducing these invasive interventions before a definitive diagnosis is difficult, genetic diagnosis during the early postnatal period is critical. In the first-born child, the genetic diagnosis performed on the 28th day after birth could be a step-wise procedure for avoiding invasive interventions and includes daily replacement of albumin with left lateral nephrectomy with PD and right nephrectomy with hybrid PD/HD and kidney transplantation. In addition, securing donors for kidney transplantation is important for children with CNF. In cases where a previous child had required a kidney transplant from either of the parents, securing a donor for the next child is very urgent. In the case of twins with CNF, as in this case, early definitive diagnosis is important for securing donors for kidney transplantation. Genetic testing of asymptomatic patients, such as for the second-born child, should be performed more carefully. It is recommended that genetic testing should be performed after the patient has a sufficient understanding of the clinical course and prognosis of the disease, its prevention, and treatment. In this case, because CNF develops in infancy and becomes fatal, and long-term management is required, an early genetic diagnosis of the second-born child was performed after frequent and in-depth discussions with the parents. Although CNF is a rare disease, its survival rate has been improved by advances in the dialysis technique for low-birth-weight infants and in kidney transplantation [9,10]. As the number of life-saving cases increases, antenatal diagnosis of second-born children is a major issue. On the other hand, in the case of twins, antenatal diagnosis is limited; therefore, strict observation and early postnatal genetic testing are key in promoting improved health outcomes.

## Conclusions

Early genetic diagnosis could clarify the risks and treatment plans for each twin. A stepwise multidisciplinary treatment plan could be developed for the affected infant, and unnecessary medical intervention could be avoided for the healthy infant. Since there are few reports of twin cases of CNF, further accumulation of cases is needed.
